# Quality of life of Moroccan patients on the palliative phase of advanced cancer

**DOI:** 10.1186/s13104-019-4390-1

**Published:** 2019-06-21

**Authors:** Aitouma Ahlam, Mrabti Hind, Bouchra Haddou Rahou, Razine Rachid, Errihani Hassan

**Affiliations:** 1 Department of Medical Oncology, National Institute of Oncology, Rabat, Morocco; 2 Translational Oncology Research Team, Faculty of Medicine and Pharmacy, Mohamed V University, Rabat, Morocco; 3 Research Department, Higher Institute of Nursing Professions and Technical Health, Rabat, Morocco; 4 Laboratory of Social Medicine and Public Health, Faculty of Medicine and Pharmacy, Mohamed V University, Rabat, Morocco

**Keywords:** Advanced cancer, Quality of life, Palliative phase, QLQ-C15-PAL

## Abstract

**Objective:**

The aim of this study is to assess the quality of life of caregiver’s. The study was conducted at the RABAT National Institute of Oncology in MOROCCO.

**Results:**

120 patients on the palliative phase of advanced cancer were included. Severe fatigue was observed in 64.2% of patients with an average of 90.55 ± 14.7. There was a positive association between functional dimensions and overall quality of life and a negative association between symptoms and overall quality of life. Patients under 30 years had a lower quality of life. According to the multi-varied analysis, physical function, emotional functioning and fatigue were significant predictors of Health related quality of life/overall quality of life (p < 0.05).

**Electronic supplementary material:**

The online version of this article (10.1186/s13104-019-4390-1) contains supplementary material, which is available to authorized users.

## Introduction

Nowadays, for many patients, the palliative phase is not equal to the terminal phase, but to a treatment phase, in which the intention is to prolong the patient’s life and/or relieve symptoms, transforming advanced cancer into a chronic disease [1]. On the other hand, when metastases have developed and cancer is in all likelihood incurable, patients find themselves in a situation of psychological fragility due to the existential threat now realized, regardless of their individual prognosis [2]. They experienced situations of hope, uncertainty, disappointment, exhaustion and, most of the time, physical deterioration [3].

Patients with advanced palliative cancer suffer from various symptoms [4]. The most distressing are Fatigue, pain and anxiety, others considered common, including lack of appetite, dyspnea, constipation and nausea [5]. Patients with advanced palliative phase cancer have a poor quality of life regardless of the type of cancer [4, 6].

Frequent occurrence of symptoms has been shown to have a significant impact on patient satisfaction with health-related quality of life (HRQoL) [7–10].

HRQoL has been defined as a state of well-being that is based on components: the ability to perform daily activities that reflect physical, psychological and social well-being; patient satisfaction with their level of functioning, control of their illness and symptoms related to their treatment [11, 12].

QoL assessment is structured around at least three dimensions, which are the physical, psychological and social domains [11, 13]. For other teams, an assessment of quality of life must be based on an additional field, symptomatology, thus allowing an assessment of quality of life in at least four dimensions [14, 15].

It is necessary to consider the particular context of advanced palliative cancer patients, including physical impairment, loss of autonomy, pain, anxiety, anger, time, feeling of being a burden, relationships with others [16].

In the lung cancer literature, this concept was explored using the EORTC QLQ-C30 questionnaire to study the relative importance of the various symptoms and functional domains of health-related quality of life in predicting overall QOL [17, 18].

Patients with advanced, palliative or terminal cancer are a sensitive, vulnerable population that requires special attention.

For that an abbreviated version of the QLQ-C30, known as QLQ-C15-PAL, was developed to reduce the burden of the longer and more cumbersome questionnaire to be completed by patients with advanced palliative cancer to assess their quality of life [19].

Several studies have shown a deteriorated quality of life of patients with advanced cancer in the palliative phase [16]. But in Morocco no study is interested in the quality of life of this category of patients.

The main objective of this study is to evaluate the quality of life of Moroccan patients with advanced palliative cancer.

The results of our study will highlight the difficulties experienced by this population to enable them to benefit from global and integrated care.

## Main text

### Methods

#### Instruments and procedures

Information about patient characteristics: age, sex, cancer location, stage, metastasis, duration of palliative phase, marital status, employment status and treatment received, were collected from medical records. GHS and QoL were assessed using QLQ-C15-PAL, it is a questionnaire that has been developed specifically for use in palliative care settings through validation studies [20–22]. This QOL measurement tool includes 15 questions; two multi-element functional scales (physical and emotional functioning), two multi-element symptomatic scales (fatigue and pain) as well as five single-element symptomatic scales (nausea/vomiting, dyspnoea, insomnia, loss of appetite, constipation) and a final question concerning general QOL.

In order to generate the scores of the different scales of the QLQ-C15-PAL, the EORTC QLQ-C30 scoring manual [23] and the QLQ-C15-PAL addendum [24] were used. Scores are defined on a scale from 0 to 100. Scores close to 100 for the symptom scale represent a high symptom load. A high score for both functional scales and the last question of the QLQ-C15-PAL, which refers to the patient’s perception of the overall QOL; represents a positive response.

The EORTC QLQ-C- 15 PAL questionnaire was translated into Moroccan dialectal Arabic, adapted and validated for Moroccan context [25].

To assess the patient’s clinical condition, the Karnofsky Performance Scale was used.

#### The subjects recruitment

The EORTC QLQ-C-15 questionnaire was administered to patients with advanced stage cancer reported in the palliative phase and not receiving chemotherapy or palliative radiotherapy who were hospitalized in the oncology and palliative care departments at the National Institute of Oncology (INO) SIDI MOHAMED IBN ABDILLAH in RABAT/MOROCCO; first reference centre in cancer treatment in Morocco.

Were included in the study, patients diagnosed with histological confirmed cancer and in palliative phase who were hospitalized in the above-mentioned services, patients of Moroccan nationality and who had signed an informed consent letter to participate in the study. Exclusion criteria are patients with psychiatric or neurological disorders that may impair understanding and adherence to the study.

Using the medical records, we identified the cancer patients who met the criteria and recruited them to participate in the study from December 2017 to July 2018.

### Statistical analysis

A descriptive analysis of the socio-demographic and clinical characteristics was carried out. We calculated means, standard deviations, minimum and maximum values. The ANOVA test was used to analyze differences in scores between sex, age groups and duration of palliative care and also to compare the average scores of the different scales to a classification of patient groups using the KPS.

Pearson correlations were calculated between all functional/symptom scales and the global scale of quality of life. A simple linear regression model was applied to detect the association between Q15 (global QOL) and functional and symptomatic dimension scores. Significant variables from the univariate analysis (p < 0.05) were introduced into the multivariate analysis.

The tests were considered significant when the p (degree of significance) was less than 0.05. The statistical analysis is performed using the SPSS version software 13.0.

## Results

Out of a total of 120 patients included in the study, 57.5% were female and 54% were illiterate. Participants were aged 20 to 92 years. The most frequent primary cancer diagnoses were respectively (43% gynaecological and mammary, 37.5% digestive, 22% pulmonary). 65.9% of patients had a Karnovsky index between 40 and 50% and the average duration of palliative phase was 22.89 months with extremes [1–204]. Other socio-demographic and clinical characteristics are presented in (Table 1).

**Table 1:** Characteristics of patient and diseases (N = 120)

	Characteristics	N	%
Age(years)	Age (mean ± SD)	120	59 ± 16.5
Gender	Female	69	57.5
	Male	51	42.5
Residence	Urban	93	77.5
	Rural	27	22.3
Marital status	Married	94	78.3
	Widower	12	10
	Single	14	11.6
Level of education	Illiterate	54	45
	Primary school	36	30
	Secondary school	28	23.4
	University	1	0.8
Employment status	Unemployed	76	73.3
	Employed	44	36.7
Social security coverage	RAMED	100	83.3
	AMO	19	15.8
Primary cancer diagnosis	Lung	22	18.3
	Digestive	45	37.5
	Gynecological and Breast	43	35.8
	Uro	5	4.2
	Others	5	4.2
Treatment received	Yes	99	82.5
	No	21	17.5
Clinical stage	M0	51	42.5
	M1	15	12.5
	NP	54	45
Karnofsky index	≤ 30	31	25.8
	[40–50]	79	65.9
	≥ 60	10	8.4
Period of transition to palliative phase (months/mean)		22.89	

AMO: insurance for public sector employees; RAMED: insurance for economically weak patients

A poor overall quality of life was reported in 32.5% of patients with an average of (24.44 ± 21.27), 46.7% of patients found their functioning extremely impaired (15.46 ± 17.18). 28.3% of participants reported that they had no problems with the emotional component and 34.2% made this component responsible for an extreme problem in their daily lives with an average score of 50.13 ± 42.03.

In our study population, 64.2% showed fatigue with an average of 90.55 ± 14.7, 20% experienced nausea/vomiting (43.88 ± 36.15), 20% experienced pain (74.30 ± 19.79), 25% experienced dyspnoea (50.55 ± 35.63), 32.5% had insomnia (68.05 ± 26.77), 58.3% lost appetite (80.83 ± 25.07), 20.8% experienced constipation (36.94 ± 39.08) (Table 2).

**Table 2:** Quality of life; functional and symptomatic scales (n = 120)

	Mean	SD	Minimum	Maximum
Functional scales				
Physical functioning	15.46	17.18	00	55.56
Emotional functioning	50.13	42.03	00	100
Symptom scales				
Fatigue	90.55	14.77	33.33	100
Nausea and vomiting	43.88	36.15	00	100
Pain	74.30	19.79	00	100
Dyspnoea	50.55	35.63	00	100
Insomnia	68.05	26.77	00	100
Appetite loss	80.83	25.07	33.33	100
Constipation	36.94	39.08	00	100
Global health status/quality of life	24.44	21.27	00	66.67

*CI*, confidence interval, *PF* physical functioning, *PE* emotional functioning, *FA* fatigue, *NV* nausea and vomiting, *PA* pain

That results showed that women have experienced dyspnoea with a p value < 0.05. There is significant variation in the age of patients, the lowest estimate of health status being for patients under 30 years of age. The emotional functional scale is significantly associated with the age of patients. Elderly subjects (> 70 years of age) were more sensitive to the symptom of pain. Symptoms (NV) and (AP) are significantly and significantly associated with the duration of the disease. KPS is significantly related to the physical functional scale of fatigue, loss of appetite (Additional file 1: Table S1).

Correlations were significant between Q15 (GHS/QOL) and functional scales with a positive score and between Q15 and symptom scale with a negative score; with the exception of constipation (Additional file 1: Table S2).

Based on QLQ-C15-PAL dimensions; in the univariate analysis, the overall QOL (Q15) shows a positive coefficient with respect to physical and emotional functioning (p < 0.05) and a negative coefficient for fatigue, pain, dyspnea, insomnia, loss of appetite (p < 0.05). According to the multi-varied analysis of QLQ-C15-PAL, physical function, emotional functioning and fatigue (p <0.05) were significant predictors of GHS/QOL (Q15) (Table 3).

**Table 3:** Univariate and multivariate linear regression analysis of all functional/Symptomatic scales with overall QOL in palliative cancer patients

Independent variable	Univariate analysis		Multivariate analysis		
	β	P value	β	[95.0% CI]	P value
PF	0.29	0.01	[0.021 to 0. 33]	0.17	*0.02*
PE	0.39	0.00	[0.32 to 0.45]	0.38	*0.00*
FA	− 0.66	0.00	[− 0.48 to − 0.09]	− 0.28	*0.004*
NV	− 0.06	0.24			
PA	− 0.49	0.00	[− 0.12 to 0.17]	0.02	0.75
Dy	− 0.13	0.01	[− 0.14 to 0.009]	− 0.06	0.08
SL	− 0.26	0.00	[− 0.001 to 0.23]	0.11	0.052
AP	− 0.18	0.01	[− 0.13 to 0.05]	− 0.04	0.36
CO	0.003	0.95			

*Dy* dyspnoea, *Sl* insomnia, *AP* appetite loss, *CO* constipation

## Discussion

This study is the first in Morocco where elements reflecting QLQ-C15-PAL were extracted from the QLQ-C30 questionnaire translated into Moroccan dialectal Arabic and analyzed for their predictive coefficients to QOL.

The results describe the onset of symptoms and impaired functioning in patients with advanced palliative stage cancer. Significant deterioration was observed in all aspects of physical and emotional functioning, with fatigue, loss of appetite, insomnia and pain being the most common symptoms experienced by patients; nausea and vomiting were considered the least problematic. Men and women reported similar levels of problems, unlike other studies that showed clear gender differences, focusing on specific symptoms, which were observed mainly insomnia (more in men) and nausea and vomiting (more frequent in women) [26–31]. Fatigue was more pronounced in all age groups in our study, similar to the results of a previous study [19]. It is known to be one of the most distressing symptoms for palliative cancer patients and negatively affects their daily lives [32–40]; its prevalence rates can reach 99% in patients with advanced and incurable cancers [34–37, 41].

Our results didn’t show differences between age groups for physical functioning, unlike other studies that showed divergent results. Indeed, The study conducted in a German population showed that physical functioning was lower in elderly subjects with higher scores for fatigue, nausea/vomiting, pain, dyspnoea, loss of appetite and constipation [42].

Overall, elderly subjects tended to report fewer problems than younger patients with more severe quality of life deterioration. Some studies have shown that older cancer patients have similar or even better HRQoL than younger patients [43]. Others have found that a lower overall HRQoL is associated with increased age, with different expectations for HRQoL in elderly patients [44].

Our results have shown, physical and emotional functioning were significant predictors of QOL, and with such a high burden of symptoms, it makes sense to identify the most important for the patient in terms of overall quality of life, so that palliative and supportive care can be adapted accordingly.

Study conducted at the National Cancer Institute of Canada showed that patients with low emotional functioning scores; have low overall quality of life scores [45].

Fatigue was the most common symptom in patients with advanced palliative cancer and can significantly worsen their QOL. Since it was the most significant predictive symptom in this study, our results are fully consistent with the results of other studies that have shown that fatigue was influenced by many factors (pain, nausea, depression, insomnia and dyspnoea) and had a significant association with general well-being [45–47].

QLQ C 15-PAL is more effective and appropriate for measuring QOL in palliative cancer patients. It is recommended to initiate studies in Morocco using this assessment tool. The poor physical condition of palliative patients means that shorter quality of life questionnaires can help reduce burden and improve data accumulation, and appropriate assessment of quality of life in patients with advanced disease can guide targeted interventions by health personnel [48].

## Conclusion

In our study, the majority of patients with advanced palliative cancer experienced a deteriorated quality of life related to the onset and persistence of certain symptoms, particularly fatigue, loss of appetite and insomnia. Emotional and physical functioning as well as fatigue was significant predictors of overall quality of life in palliative patients.

It is recommended that accurate quality of life assessments be conducted in palliative research. Care should be based on the intensity of symptoms, their burden and their impact on patients’ quality of life.

## Limitations

Some limits must be taken into account: The QLQ C-15-PAL is a self-administered questionnaire, the problem of patient’s illiteracy, which has resulted in administration by the same investigator. Also, the short form of EORTC C 15-PAL is unable to evaluate some areas of QLQ-C-30.

### Additional file

**Additional file 1: Table S1.** Comparisons (mean ± standard deviation) and correlations between quality of life; functional and symptomatic scales of EOTC C-15-PAL with patients’ characteristic. **Table S2.** Correlation between all functional scale variables/Symptoms and Q15 in patients with advanced palliative phase cancer.

## Glossary

### EORTC C 15 PAL

The EORTC QLQ-C15-PAL is an abbreviated 15-item version of the EORTC QLQ-C30 (version 3.0) developed for palliative care. The QLQ-C15-PAL is recommended for use in patients with advanced, incurable, and symptomatic cancer with a median life expectancy of a few months. It is not recommended for patients receiving palliative, anti-cancer treatments including chemotherapy, radiotherapy, endocrine treatments, or palliative surgery [20, 22].

### The Karnofsky Performance Scale

It is a one-dimensional, single-element functional state scale used to obtain an overall measure of activity level, particularly for patients undergoing cancer treatment. The level of functionality is assessed by a health care provider as a percentage ranging from 100% (normal, no complaints, and no signs of illness) to 0% (death) [49].

### Quality of life/global health status

Is defined as the perception that an individual has of his or her place in life, in the context of the culture and value system in which he or she lives, in relation to his or her objectives, expectations, norms and concerns. It is a very broad concept that can be influenced in a complex way by the subject’s physical health, psychological state and level of independence, social relationships and relationship to the essential elements of his or her environment [50].

Health-related quality of life is also defined as “a state of well-being that is based on two components: (1) the ability to perform daily activities that reflect physical, psychological and social well-being; (2) patient satisfaction with their level of functioning, control of their disease and symptoms related to their treatment [11, 12].

## Abbreviations

PF: physical functioning
PE: emotional functioning,
FA: fatigue
NV: nausea and vomiting
PA: pain
Dy: dyspnoea
SL: insomnia
AP: appetite loss
CO: constipation
GHS/QoL: global health status/quality of life
HDR: out of all therapeutic resources
CI: confidence interval
KPS: Karnofsky Performance Scale

## Availability of data and materials

All data generated or analysed during this study are included in this published article.
