# Supplementary material for: Quality of life of Moroccan patients on the palliative phase of advanced cancer
Source: BMC Res Notes. 2019 Jun 21;12:351. doi: 10.1186/s13104-019-4390-1 (PMC6588852; doi:10.1186/s13104-019-4390-1)
Supplement: Supplementary file 1 — Additional file 1: Table S1. Comparisons (mean ± standard deviation) and correlations between quality of life; functional and symptomatic scales of EOTC C-15-PAL with patients’ characteristic. Table S2. Correlation between all functional scale variables/Symptoms and Q15 in patients with advanced palliative phase cancer. [file 13104_2019_4390_MOESM1_ESM.docx]

**Table S1 .Comparisons (mean ± standard deviation) and correlations between quality of life; functional and symptomatic scales of EOTC C-15-PAL with patients’ characteristic**

|  | **PF** | **EF** | **FA** | **NV** | **PA** | **DY** | **SL** | **AP** | **CO** | | **QL** |
| --- | --- | --- | --- | --- | --- | --- | --- | --- | --- | --- | --- |
| **Patient’s gender** | | | | | | | | | | | |
| Women | 14 .86±17.65 | 46.81±41.35 | 91.17±15.63 | 42.15±36.70 | 75.00±19.84 | 45.58±35.45 | 70.58±27.33 | 77.94±25.50 | 32.35±35.95 | 22.79±22,47 | |
| Men | 16.44±16.83 | 54.66±42.59 | 89.66±13.83 | 46.00±36.19 | 73.33±19.63 | 58.00±35.51 | 64.66±25.56 | 85.33±24.43 | 42.66±42.07 | 26.33±19.65 | |
| P value | 0.5 | 0.3 | 0.2 | 0.4 | 0.1 | **0.04** | 0.1 | 0.1 | 0.3 | 0.5 | |
| **Patient’ age class(years)** | | | | | | | | | | | |
| ˂ 30 | 22.22±25.66 | 21.42±36.91 | 88.09±24.93 | 19.04±37.79 | 78.57±15.85 | 28.57±40.49 | 66.66±19.24 | 85.71±17.81 | 19.04±37.79 | 11.90±24.93 | |
| 30-70 | 14.96±16.28 | 40.00±40.26 | 92.44±12.64 | 46.66±35.51 | 77.77±17.40 | 52.44±33.85 | 69.33±25.56 | 82.22±25.31 | 39.55±39.39 | 19.77±18.72 | |
| ˃70 | 15.20±17.41 | 75.43±34.60 | 87.28±16.17 | 42.98±36.27 | 66.66±22.92 | 50.87±37.75 | 65.78±30.49 | 77.19±25.82 | 35.08±38.70 | 35.96±21.05 | |
| P value | 0.5 | **0.00** | 0.1 | 0.1 | **0.01** | 0.2 | 0.7 | 0.5 | 0.3 | **0.00** | |
| **Patient’s disease duration (months)** | | | | | | | | | | | |
| ˂ 12 | 12.80±17.11 | 55.36±40.99 | 92.09±14.63 | 49.71±37.84 | 71.18±21.40 | 50.84±36.28 | 68.92±27.58 | 88.13±21.22 | 41.80±41.33 | 24.57±20.84 | |
| 12-48 | 17.57±17.69 | 51.55±43.91 | 87.20±14.92 | 43.41±34.53 | 75.96±17.92 | 52.71±37.95 | 65.89±26.71 | 77.51±26.94 | 30.23±35.49 | 25.58±22.52 | |
| ˃48 | 19.25±15.97 | 33.33±38.31 | 92.22±15.25 | 17.77±21.33 | 78.88±18.32 | 37.77±21.33 | 64.44±23.45 | 60.00±22.53 | 33.33±41.78 | 23.33±20.70 | |
| P value | 0.2 | 0.1 | 0.2 | **0.08** | 0.2 | 0.3 | 0.7 | **0.00** | 0.3 | 0.9 | |
| **Karnovsky performance score(KPS)** | | | | | | | | | | | |
| 30 | 7.88 ±14.39 | 62.90±39.37 | 94.08±15.83 | 47.31±38.27 | 75.80±19.16 | 55.91±35.88 | 74.19±28.16 | 83 .87±24.14 | 30.10±36.87 | 29.03±22.34 | |
| 40 | 16.38 ± 16.02 | 50.00±42.88 | 89.54±12.34 | 48.02±36.76 | 73.44±18.34 | 49.15±34.10 | 65.53±24.73 | 83.61±23.46 | 42.37±41.44 | 25.98±20.35 | |
| 50 | 17.22±15.90 | 40.83±39.54 | 91.66±14.80 | 33.33±30.58 | 72.50±24.34 | 48.33±42.54 | 68.33±31.48 | 70.00±28.40 | 28.33±34.66 | 15.83±17.50 | |
| 60 | 27.16±23.64 | 33.33±44.09 | 87.03±20.03 | 33.33±33.33 | 79.62±23.24 | 48.14±33.79 | 66.66±23.57 | 81.48±24.21 | 40.74±40.06 | 18.51±28.19 | |
| P value | **0.002** | 0.1 | **0.03** | 0.2 | 0.8 | 0.8 | 0.4 | **0.05** | 0.4 | 0.2 | |

PF : Physical functioning, PE : Emotional functioning, FA : Fatigue, NV : Nausea and vomiting, PA : Pain Dy : Dyspnoea,SL: Insomnia, AP : Appetite loss, CO : Constipation

**Table S2. Correlation between all functional scale variables/Symptoms and Q15 in patients with advanced palliative phase cancer**

| variable | Correlation (r ) | P-Value¹ | N |
| --- | --- | --- | --- |
| Physical functioning | 0.23 | 0.01 | 120 |
| Emotional functioning | 0.78 | 0.00 | 120 |
| Fatigue | -0.46 | 0.00 | 120 |
| Nausea and vomiting | -0.10 | 0.24 | 120 |
| Pain | -0.46 | 0.00 | 120 |
| Dyspnoea | -0.23 | 0.01 | 120 |
| Insomnia | -0.33 | 0.00 | 120 |
| Appetite loss | -0.22 | 0.01 | 120 |
| Constipation | 0.05 | 0.5 | 120 |

1. All p-values are less than 0.05 and considered statistically significant. QVG : Global health status/Quality of life
